# Thiosinamin

**Published:** 1898-02

**Authors:** 


### THIOSINAMIN.

In the *New York Medical Journal* of November 6, 1897, appears a paper second to that published in May, 1896, by Dr. Sinclair Tousey, of New York, detailing further experience with Thiosinamin in the treatment of keloid, inoperable tumors and cicatricial conditions generally.

This drug, a derivative of mustard-oil, chemically allied to urea, was first studied by Hebra, Keitel, Richter, Van Horn and others, and tested in the treatment of tuberculosis. While failing entirely in this affection it was incidentally observed to have a markedly beneficial effect on cicatricial deformities following lupus, and several cases of ectropion were thus cured. These and other clinical results, together with the fact that its hypodermic administration is followed by a rapid and intense leucocytosis suggested a trial of its action in keloid, in which condition according to the author's experience it has proved uniformly curative. At the present time it is, the author states, in routine use in the Vienna clinics for this affection. Its range of usefulness does not seem to be confined, however, to the dissipation of keloid growths. A number of reports are now recorded of its solvent action on scar-tissue from various causes; it appears to affect in a marked degree the absorption of adventitious low-grade tissues and post-inflammatory deposits. Hebra, a year ago, reported the cure of a number of cases of corneal opacity by its use at the Rudolfspital of Vienna. This is also the experience of Richter. It seems best adapted in this regard to cases of slight or medium severity, and care must be taken not to institute treatment until inflammatory action has completely subsided. In the present paper the author urges a trial of this drug in deafness due to thickening of the tympanic membrane.

In the treatment of urethral stricture this remedy has met with gratifying success. "It may enable us," says Dr. Tousey, "by preparatory treatment to convert a difficult and dangerous operation into a simple one." Thiosinamin may be used by mouth or hypodermically, and preferably by the latter method. A solution is made by dissolving ten parts of the drug in a hundred parts of a sterile mixture of water and glycerine, which keeps well and is non-irritant. As a full dose twelve or fifteen minims are injected into a muscle, triceps or gluteus, every three days. In each case the amount must be adjusted to the patient, but the most useful dose in the author's experience is the above.—*Cleveland Journal of Medicine.*

